# A deep learning model, NAFNet, predicts adverse pathology and recurrence in prostate cancer using MRIs

**DOI:** 10.1038/s41698-023-00481-x

**Published:** 2023-12-11

**Authors:** Wei-jie Gu, Zheng Liu, Yun-jie Yang, Xuan-zhi Zhang, Liang-yu Chen, Fang-ning Wan, Xiao-hang Liu, Zhang-zhe Chen, Yun-yi Kong, Bo Dai

**Affiliations:** 1 Department of Urology, Fudan University Shanghai Cancer Center, Shanghai, China; 2 Department of Oncology, Shanghai Medical College, Fudan University, Shanghai, China; 3 Shanghai Genitourinary Cancer Institute, Shanghai, China; 4 Department of Foundation Model, MEGVII Technology, Beijing, China; 5 Department of Radiology, Fudan University Shanghai Cancer Center, Shanghai, China; 6 Department of Pathology, Fudan University Shanghai Cancer Center, Shanghai, China

**Keywords:** Prostate cancer, Cancer imaging

## Abstract

We aimed to apply a potent deep learning network, NAFNet, to predict adverse pathology events and biochemical recurrence-free survival (bRFS) based on pre-treatment MRI imaging. 514 prostate cancer patients from six tertiary hospitals throughout China from 2017 and 2021 were included. A total of 367 patients from Fudan University Shanghai Cancer Center with whole-mount histopathology of radical prostatectomy specimens were assigned to the internal set, and cancer lesions were delineated with whole-mount pathology as the reference. The external test set included 147 patients with BCR data from five other institutes. The prediction model (NAFNet-classifier) and integrated nomogram (DL-nomogram) were constructed based on NAFNet. We then compared DL-nomogram with radiology score (PI-RADS), and clinical score (Cancer of the Prostate Risk Assessment score (CAPRA)). After training and validation in the internal set, ROC curves in the external test set showed that NAFNet-classifier alone outperformed ResNet50 in predicting adverse pathology. The DL-nomogram, including the NAFNet-classifier, clinical T stage and biopsy results, showed the highest AUC (0.915, 95% CI: 0.871–0.959) and accuracy (0.850) compared with the PI-RADS and CAPRA scores. Additionally, the DL-nomogram outperformed the CAPRA score with a higher C-index (0.732, *P* < 0.001) in predicting bRFS. Based on this newly-developed deep learning network, NAFNet, our DL-nomogram could accurately predict adverse pathology and poor prognosis, providing a potential AI tools in medical imaging risk stratification.

## Introduction

Prostate cancer was the second most frequently diagnosed cancer and the fifth leading cause of cancer-specific death among men in 2020. With an estimated 1.4 million new cases diagnosed worldwide and over 375,000 deaths attributed to this disease annually^1^. Notably, approximately 29% of patients encounter Gleason grade upgrading, and 25% experience adverse pathology following radical prostatectomy, potentially leading to biochemical recurrence or the development of metastasis^2,3^. Addressing the challenges associated with adverse pathology (AP) and biochemical recurrence survival (BCR) prediction is vital for enhancing the precision and efficacy of prostate cancer management strategies.

Multiparametric magnetic resonance imaging (mpMRI) plays important roles in diagnosing high-grade or clinically significant prostate cancer (csPCa), planning for definitive treatment and predicting the presence of AP, such as seminal invasion^4,5^. Integrating mpMRI with clinicopathologic parameters may allow for better risk classification of prostate cancer before radical surgery or radiation^6^.

AP is known as an important predictor of BCR and metastasis^7^. Recently, several studies have indicated that radiomic features derived from biparametric MRI, which evaluates subvisual texture patterns for the quantitative characterization of tumour phenotypes, might aid in prostate cancer risk stratification^8–11^. However, these systems require considerable effort in carefully ruling out the features before training a machine learning classifier, and suffering limitation of relatively low efficiency. Therefore, there is a high medical need for faster procedures.

One possible solution could be the use of pretreatment parameters including clinical characteristics and MRI images to predict AP after radical prostatectomy (RP). Convolutional neural networks have successfully been used to analyse urological medical images in the fields of cystoscopy, ultrasound, pathology, and radiology^12,13^. One of the most widely used algorithms in computer vision, ResNet50, has been shown its potent potential in medical imaging. However, with 50 convolutional layers, ResNet50 needs substantial resource for further analyses^14^. The interpretation and classification of MRI rely on the understanding of each pixel in the MRI image. Since the lesion area occupies a small proportion in the image, these pixels must be fully utilized by the model to make accurate diagnoses. We here introduce NAFNet, a nonlinear activation-free network that simplifies the architecture and replacing the complex nonlinear activation functions. Although simplified, NAFNet still achieved state-of-the-art results with lower computational costs^15,16^. Moreover, the pixelwise feature segmentation capability of NAFNet enables it to extract and identify subtle differences in medical imaging, thus making it promising in medical imaging classification, especially in MRI.

In summary, within the present study, we aimed to explore the use of a deep learning algorithm (NAFNet) to identify AP on MRI image, and compare its performance with ResNet50. We next developed a customed nomogram by integrating this classifier and pretreatment clinical variables to predict AP and BCR events. We then clinically validated its performance in external cohorts by comparing it to current benchmark models such as preoperative Cancer of the Prostate Risk Assessment (CAPRA) nomograms and PI-RADS score.

## Results

### Patients characteristics

**Fig. 1:**
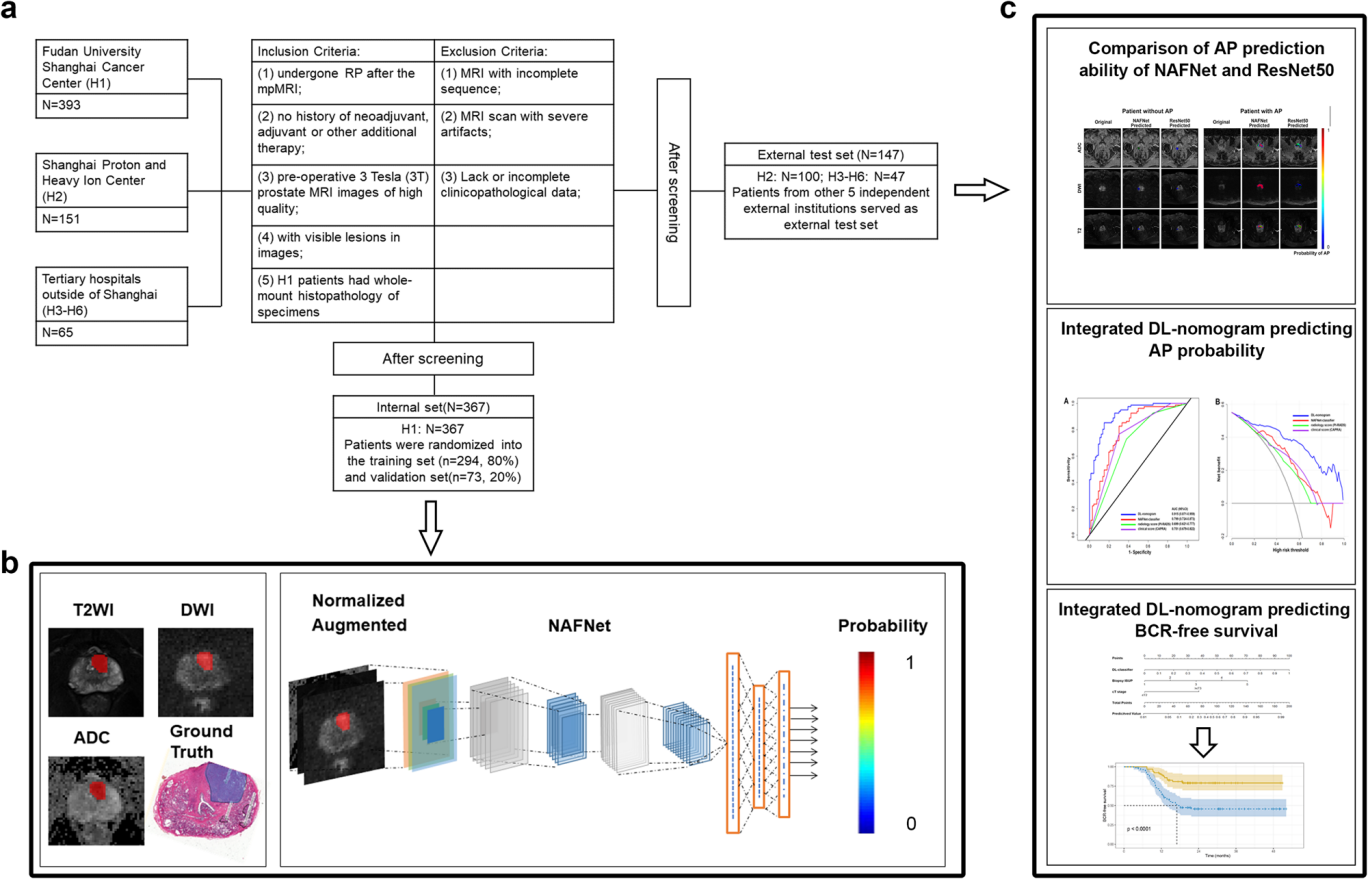
Overall flowchart and workflow of this study. **a** Flowchart of study population selection showing the inclusion and exclusion criteria for six hospitals. **b** Work pipeline of this study illustrating the construction of the deep-learning model based on three input MRI channels using internal set data. **c** After training, we used external dataset to compare the ability of the NAFNet-classifier and the ResNet50-classifier to predict adverse pathology (top panel); we also evaluated the predictive ability of the integrated DL-nomogram for adverse pathology (middle panel) and for BCR-free survival (bottom panel). Abbreviations: RP radical prostatectomy, mpMRI multiparametric magnetic resonance imaging, T2WI T2-weighted magnetic resonance imaging, DWI diffusion-weighted imaging, ADC apparent diffusion coefficient, AP adverse pathology, DL deep learning, BCR biochemical recurrence.

We retrospectively enrolled 618 patients with prostate cancer from six institutes throughout China. As depicted in Fig. 1a, after screening, 367 eligible patients from Fudan University Shanghai Cancer Center were allocated to the internal set. The external test set consisted of 147 patients from other five external institutes. Patient characteristics were listed in Table 1, and there were no statistically significant differences between clinicopathological variables of the internal and external set. The NAFNet network was trained to use the labelled images from the internal set to predict the AP probability of each patient (Fig. 1b). After training and validation, we applied the best model to the external test set to compare NAFNet with ResNet50 for predicting AP, to construct DL-nomogram, and to evaluate its AP and bRFS prediction ability (Fig. 1c). Of note, patients in the external test set did not have whole-mount histopathology, and tumour lesions were identified by radiologists who were blinded to the clinical and pathological information.

**Table 1:** Patient baseline characteristics.

Factor	Internal Set (*n* = 367)		External Test Set (*n* = 147)		*P*-value^a^
	No.	%	No.	%	
Age at surgery (year), median (IQR)	67 (62–72)		67 (63–72)		0.771^b^
PSA at diagnosis (ng/ml), median (IQR)	12.08 (7.21–22.80)		13.00 (8.48–25.67)		0.261^b^
PSA at diagnosis (ng/ml)					0.873
≤20	252	68.7	102	69.4	
>20	115	31.3	45	30.6	
Clinical T stage					0.370
cT2	319	86.9	132	89.8	
≥cT3	48	13.1	15	10.2	
Biopsy ISUP					0.652
1	79	21.5	27	18.4	
2	64	17.4	20	13.6	
3	60	16.3	26	17.7	
4	102	27.8	44	29.9	
5	62	16.9	30	20.4	
PI-RADS v2 score					0.655
3	60	16.3	29	19.7	
4	87	23.7	34	23.1	
5	220	60.0	84	57.1	
Post-surgery ISUP					0.407
1	31	8.4	13	8.8	
2	115	31.3	35	23.8	
3	97	26.4	48	32.7	
4	49	13.4	23	15.6	
5	75	20.4	28	19.0	
Extra-prostatic extension					0.105
Present	122	33.2	60	40.8	
Absence	245	66.8	87	59.2	
Surgical margin status					0.362
Positive	44	12.0	22	15.0	
Negative	323	88.0	125	85.0	
Seminal vesicle invasion					0.280
Present	67	18.3	21	14.3	
Absence	300	81.7	126	85.7	
Lymph node metastasis					0.603^c^
Present	10	2.7	6	4.1	
Absence	357	97.3	141	95.9	
Adverse pathologic events					0.159
Present	177	48.2	81	55.1	
Absence	190	51.8	66	44.9	
CAPRA score					0.349
1–2	34	9.3	11	7.5	
3–5	154	41.9	54	36.7	
6–10	179	48.8	82	55.8	
Biochemical recurrence events					<0.001
Present	19	5.2	56	38.1	
Absence	348	94.8	91	61.9	

^a^Chi-square test was applied unless otherwise stated.

^b^Mann–Whitney *U*-test.

^c^Continuity Correction.

*IQR* interquartile range, *PSA* Prostate specific antigen, *PI-RADS* Prostate Imaging–Reporting and Data System, *ISUP* International Society of Urological Pathology, *CAPRA* Prostate Cancer Risk Assessment.

### NAFNet is superior to ResNet50 in predicting AP based on MRI imaging

**Fig. 2:**
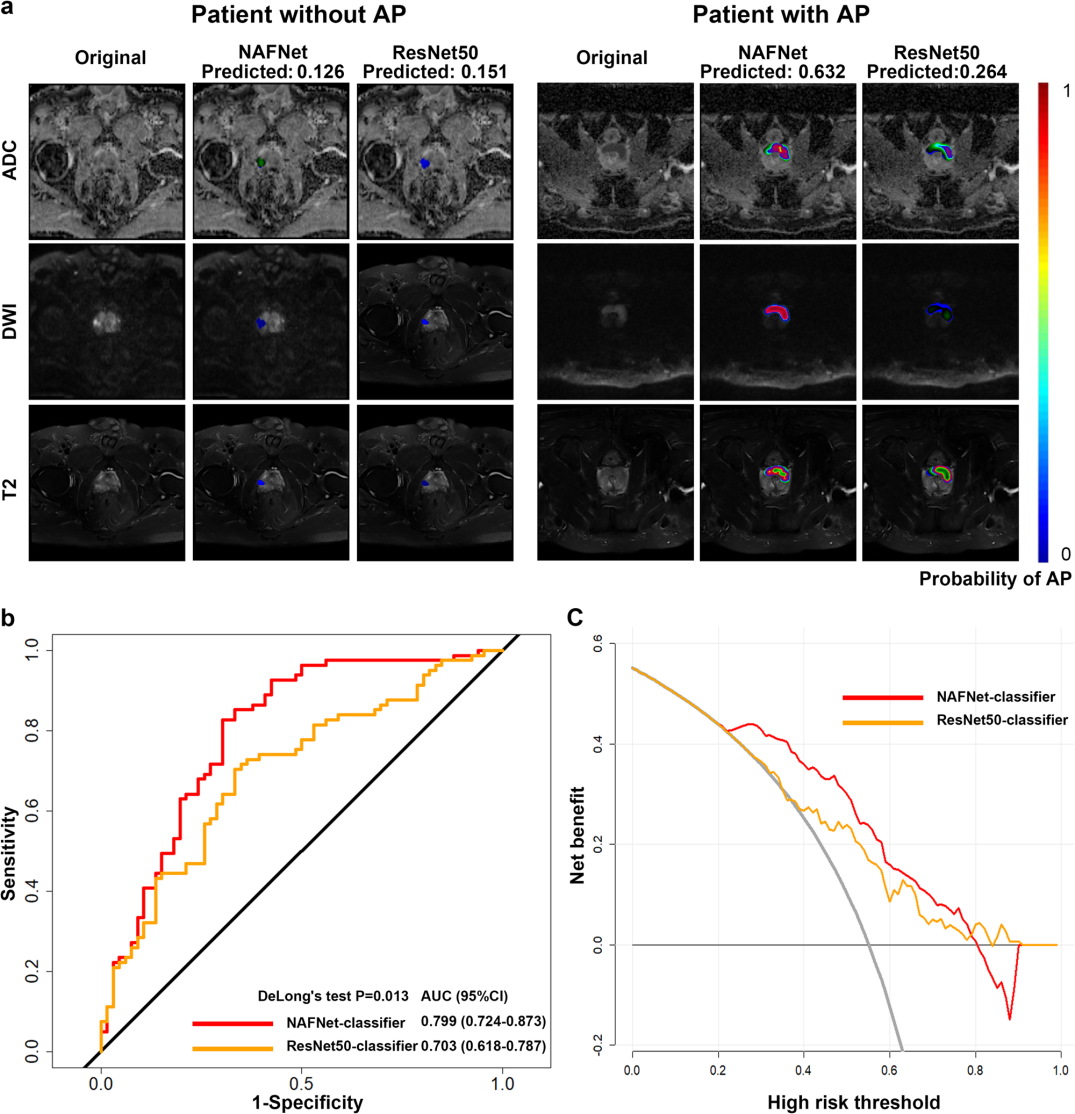
Comparison of the predictive ability for adverse pathology between the NAFNet-classifier and the ResNet50-classifier. **a** NAFNet-classifier and ResNet50-classifier prediction visualized results on the MRI image depicting the probability of adverse pathology in identified regions. The left panels show MRI images from a patient who did not experience any adverse pathology events. The NAFNet-classifier predicted an AP probability of 0.126, while the ResNet50-classifier predicted a probability of 0.151. While the right panels depict MRI images from a patient with an adverse pathology event. The NAFNet-classifier gave a 0.632 AP probability, and the ResNet50-classifier had a probability of 0.264. The colour scale on the right represents the probability of adverse pathology events in each pixel within the identified region, with red showing a higher probability; and blue showing a lower probability. **b** Receiver operating characteristics curves and (**c**) decision curve analyses of NAFNet-classifier and ResNet50-classifier in predicting adverse pathology events based on external test set. Abbreviations: AP adverse pathology, ADC apparent diffusion coefficient T2WI, DWI diffusion-weighted imaging, T2-weighted magnetic resonance imaging, AUC area under the receiver operating characteristic curve, CI confidence interval.

To explore the algorithmic advantages of NAFNet, we compared the NAFNet with ResNet50, a most widely-used network in computer vision and medical imaging deep learning. Both NAFNet-classifier and ResNet50-classifier prediction visualized results on the MRI image were illustrated in Fig. 2a. The blue area indicates the lowest probability of AP events, while the red area represents the highest risk of AP events. The left panels were from a patient without AP events, and the right panels were from a patient with confirmed AP events. The visualization results of NAFNet-classifier and ResNet50-classifier are quite different, especially in predicting patients with AP event (Fig. 2a, right panel). We then analysed the ROC curves between these two models on the external test set. As shown in Fig. 2b, compared with ResNet50-classifier (AUC: 0.703, 95%CI:0.618-0.787), NAFNet-classifier had a significant higher AUC value (0.799, 95%CI:0.724−0.873; *P* = 0.013). Further DCA analyses also indicated that NAFNet-classifier could provide greater benefits than ResNet50-classifier (Fig. 2c), indicating NAFNet surpassed ResNet50 in predicting AP from MRI images. Detailed performance comparison metrics were also listed in Supplementary Table 1, NAFNet-classifier had better accuracy, sensitivity and specificity than ResNet-50 classifier.

Furthermore, to compared NAFNet-classifier with ResNet50-classifier on the binary classification setting, we also created confusion matrices of these two models. The optimal threshold of dichotomous classification was listed in Supplementary Table 1. As illustrated in Supplementary Fig. 1, even when picking a threshold to create the classes, the NAFNet-classifier still outperformed the ResNet50-classifier with higher accuracy, positive prediction value, and negative prediction value (Supplementary Table 2).

### Construction of the deep-learning based nomogram

**Fig. 3:**
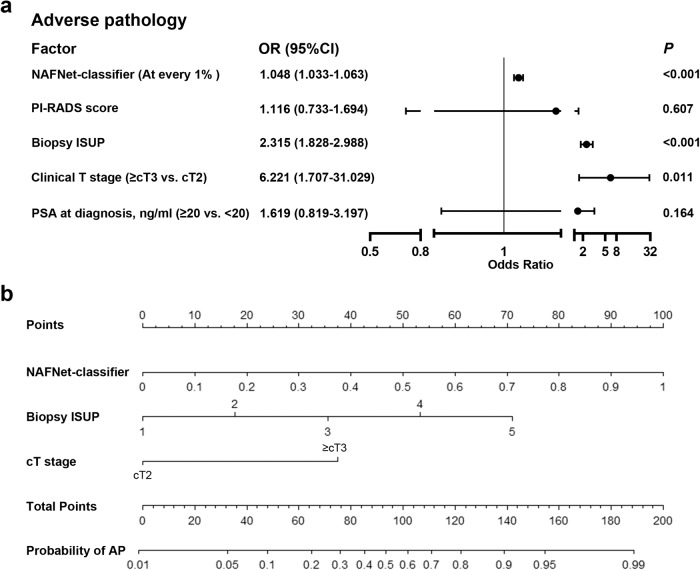
Establishment of the DL-nomogram, a nomogram integrating NAFNet-classifier and clinical variables. **a** Forest plot shows the multivariate logistic regression analyses constructed based on internal set for predicting adverse pathology events. NAFNet-classifier was treated as continuous variable. **b** A nomogram combining the NAFNet-classifier, biopsy ISUP and clinical T stage. Abbreviations: DL deep learning, PI-RADS Prostate Imaging–Reporting and Data System, ISUP International Society of Urological Pathology, PSA prostate specific antigen, OR odds ratio, CI confidence interval, AP adverse pathology.

To further establish the deep learning-based nomogram, we sought to combine the NAFNet-classifier with other clinicopathological variables. We then performed multivariate logistic regression analyses to determine which clinical variables should be integrated with NAFNet-classifier. As shown in Fig. 3a, the NAFNet-classifier (as continuous variable), clinical T stage and biopsy ISUP were significant predictors for AP events, while the PI-RADS score and PSA value did not show adequate predictive ability. Thus, we constructed our integrated clinical variables nomogram (DL-nomogram) based on the NAFNet-classifier, clinical T stage and biopsy ISUP. The DL-nomogram was illustrated in Fig. 3b, indicating a high weight of the NAFNet-classifier in the DL-nomogram, and a further calibration plot showed good performance of the DL-nomogram (Supplementary Fig. 2).

In addition, we performed another logistic regression analysis considering the NAFNet-classifier as dichotomous factor using the optimal threshold as the cut-off point. As illustrated in Supplementary Fig. 3, ROC analyses results suggested that continuous NAFNet-classifier has advantages in AUC than dichotomous NAFNet-classifier. Thus, we treated NAFNet-classifier as a continuous variable in further analyses.

Although the PSA value and the PI-RADS score did not have statistical significance in logistic regression model (Fig. 3a), they did serve as important variables to guide treatment and follow-up in prostate cancer. As a supplement to nomogram construction, we also built nomograms including PSA and PI-RADS (Supplementary Fig. 4a–c). Nevertheless, we found that adding PSA and/or PI-RADS to DL-nomogram did not further improve the efficiency of DL-nomogram in both ROC and DCA analyses (Supplementary Fig. 4d, e and Supplementary Table 2).

### Performance comparisons of deep learning-based models to predict AP events

**Fig. 4:**
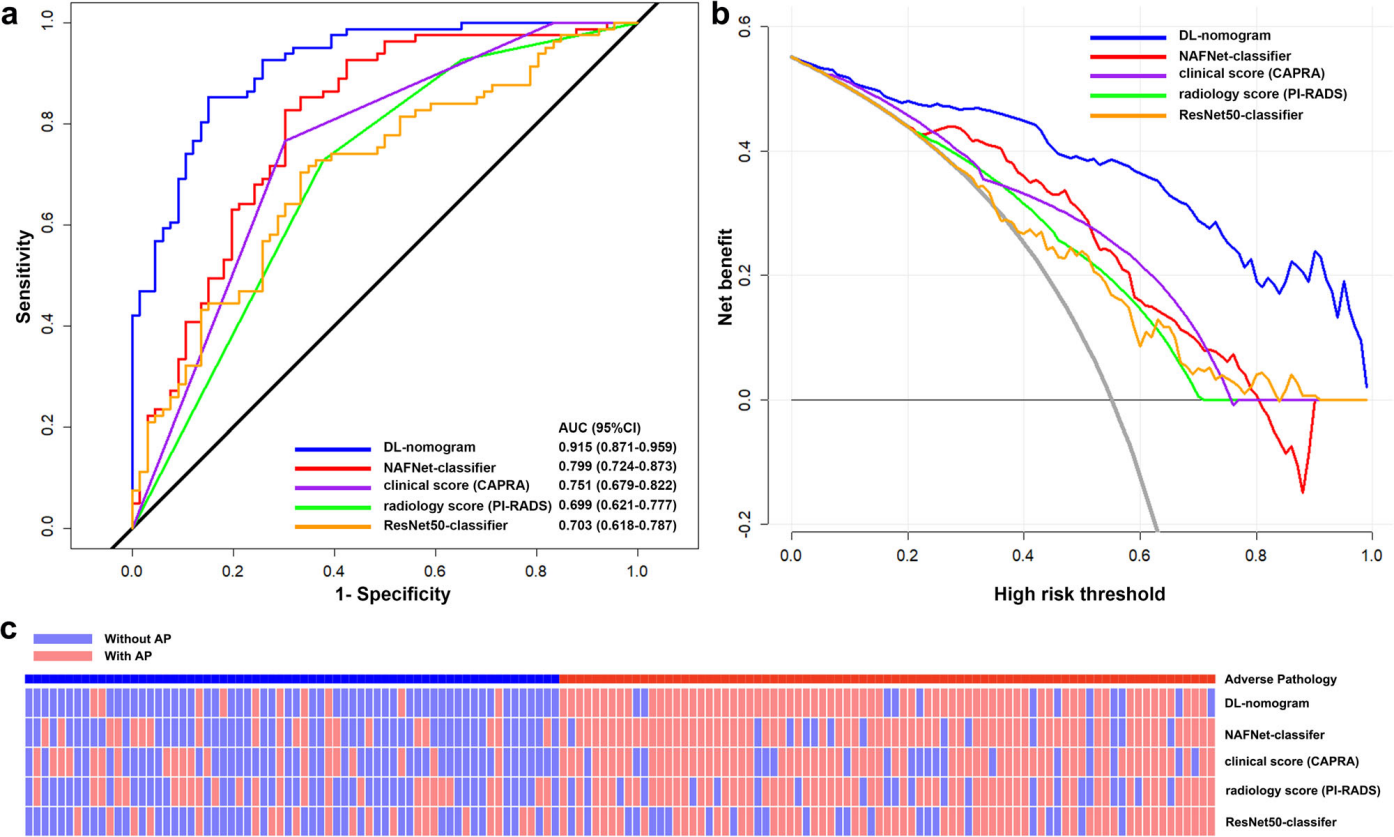
Performance analysis of various models in predicting adverse pathology events. **a** Receiver operating characteristics curves and (**b**) decision curve analyses of the DL-nomogram, NAFNet-classifier, radiology score (PI-RADS), clinical score (CAPRA) and ResNet50-classifier in predicting adverse pathology events. **c** Heatmap visualizing the prediction outcome of DL-nomogram, NAFNet-classifier, radiology score (PI-RADS), clinical score (CAPRA) and ResNet50-classifier in predicting adverse pathology events. Abbreviations: AUC area under the receiver operating characteristic curve, CI confidence interval, DL deep learning, CAPRA Prostate Cancer Risk Assessment, PI-RADS Prostate Imaging–Reporting and Data System.

To validate the performance of the deep learning-based models, we evaluated the AP prediction ability of the NAFNet-classifier, DL-nomogram, clinical score (CAPRA), radiology score (PI-RADS) and ResNet50-classifier on the external test set. As shown in Fig. 4a and Supplementary Table 1, while the AUC differences between the clinical score (CAPRA) and NAFNet-classifier did not reach statistical significance (*P* = 0.334), DL-nomogram had a significantly higher AUC value than the clinical score (CAPRA) (*P* < 0.001). Besides, DL-nomogram had the highest AUC value (0.915, 95% CI: 0.871-0.959) among these five models, and also had the highest accuracy value of 0.850. DCA further showed an improved net benefit brought by DL-nomogram compared with other models (Fig. 4b).

According to the calculated threshold values listed in Supplementary Table 1, we visualized the prediction results of the five models in Fig. 4c. The heatmap clearly showed that DL-nomogram prediction results had the best accordance with the gold standard (top panel), followed by NAFNet-classifier, and then radiology score (PI-RADS), clinical score (CAPRA) and ResNet50-classifier.

### Comparisons of our NAFNet-classifier and DL-nomogram with other predicting models for predicting bRFS

**Fig. 5:**
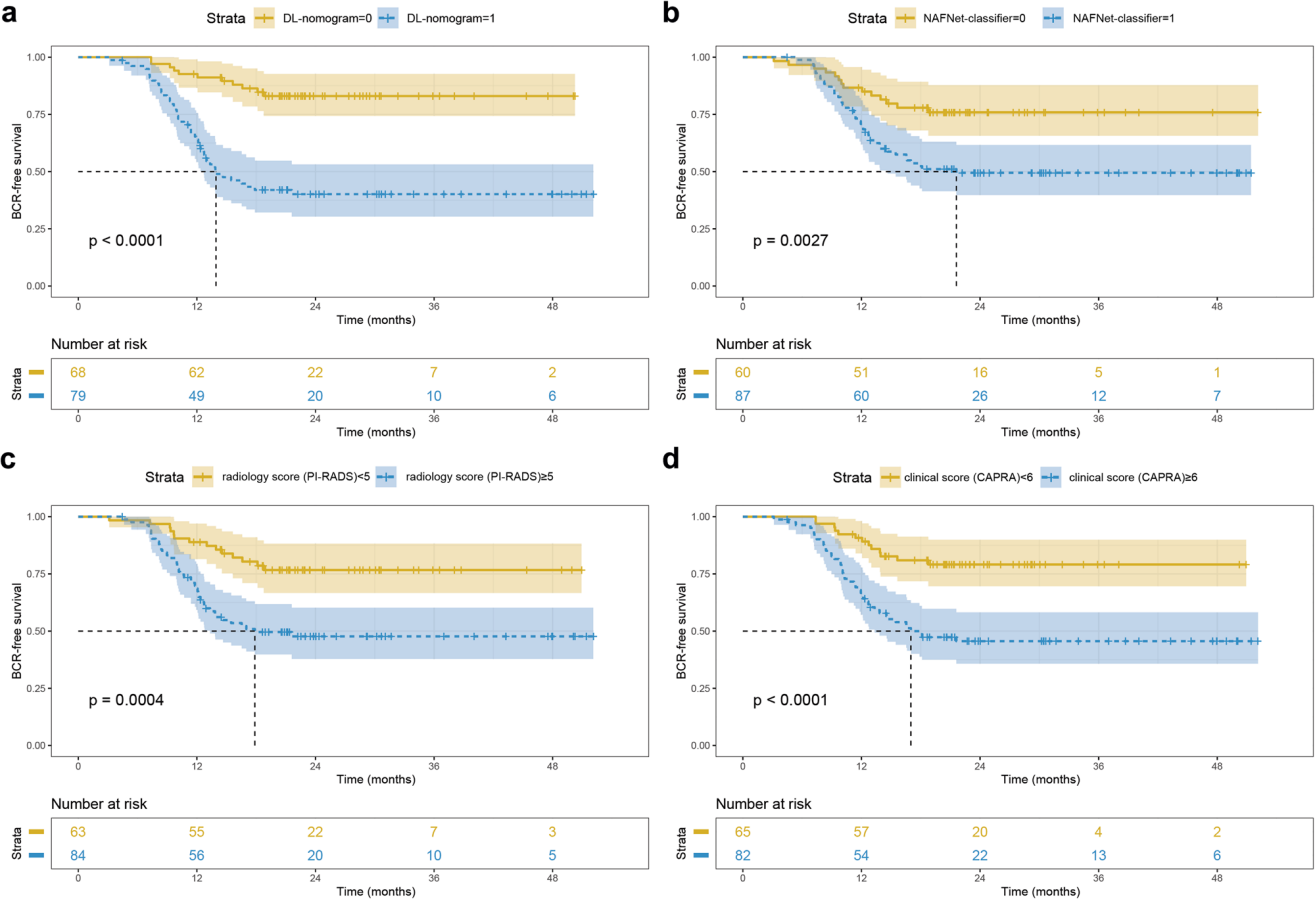
Kaplan–Meier analyses of different models in predicting biochemical recurrence-free survival. Kaplan–Meier survival curves for biochemical recurrence-free survival according to the DL-nomogram (**a**) NAFNet-classifier (**b**) radiology score (PI-RADS) (**c**) and clinical score (CAPRA) (**d**). The cut-off points of various models were determined from the optimal threshold points on the corresponding receiver operating characteristic curves of each model. Abbreviations: DL deep learning, PI-RADS Prostate Imaging–Reporting and Data System, CAPRA Prostate Cancer Risk Assessment.

To fully explore the predictive potential of the NAFNet-classifier-based model, we assessed NAFNet-classifier in BCR-free survival prediction. In the external test set, we performed Kaplan‒Meier analyses of bRFS according to the NAFNet-classifier, radiology score (PI-RADS), DL-nomogram and clinical score (CAPRA) score. The cut-off value of each model was determined by its optimal threshold in ROC curves. As illustrated in Fig. 5, all four models could stratify BCR-free survival for the external test set patients. The DL-nomogram even increased the C-index value from 0.643 (95%CI: 0.586-0.700) (CAPRA score) to 0.732 (95%CI: 0.671-0.793), showing prognostic prediction potential in BCR-free survival (Supplementary Table 3).

### Our NAFNet-classifier and DL-nomogram could also identify clinically significant prostate cancer prebiopsy

Since an ISUP score over 4 is one of the criteria of AP, we aimed to extend the NAFNet-classifier in predicting clinically significant prostate cancer before biopsy. Multivariate logistic regression analyses confirmed that the NAFNet-classifier, clinical T stage and PSA value had the ability to predict the post-surgical ISUP high-risk group (Supplementary Table 4). The DLPT-nomogram (NAFNet-classifier integrated with PSA and cT stage) was then established and had a higher post-surgical ISUP high-risk predicting performance than the clinical score (PBCG) (*P* = 0.003). Additionally, NAFNet-classifier alone also outperformed the PBCG score in both ROC analysis and DCA (Supplementary Fig. 5 and Supplementary Table 5).

## Discussion

In this study, we aimed to evaluate the capabilities of a deep learning model, NAFNet, to the pre-treatment mpMRI imaging. Because of its pixelwise segmentation capability, NAFNet-classifier had a better performance than traditional deep learning models, such as ResNet50 in predicting AP events. NAFNet-classifier not only showed good performance in predicting AP by itself, but also greatly improve the prediction ability when adding other important clinical variables, such as clinical T stage and ISUP group. Although the DL-nomogram was designed to predict AP, our multicentre results also suggest that our nomogram can triage patients with early BCR, since it has better performance than the CAPRA risk calculator. Our results thus indicated the promising potential for the DL-nomogram, as a noninvasive, fast, low-cost and accurate artificial intelligence tool, in predicting poor prognosis. The ability to accurately predict adverse prognosis in prostate cancer patients can facilitate the precise identification of individuals unsuitable for active surveillance and those who may benefit from adjuvant treatment.

As far as we know, our nomogram represents the pioneering effort in utilizing deep learning predictions to identify post-surgical AP on mpMRI images. Most previous studies used deep learning methods to automatically distinguish between clinically significant and clinically insignificant prostate cancer^17^. However, these studies used low-quality radiology images with low b-values, with the labels derived from pathology results from biopsy, which are not actually as accurate as surgical specimens.

Hiremath et al. trained deep learning-based biomarkers using AlexNet and constructed an integrated nomogram using PSA, prostate volume, lesion volume, PI-RADS score, and deep learning predictions to predict clinically significant prostate cancer lesions, as well as bRFS^17^. Li et al. screened radiomic features and developed a nomogram, including radiomics-based imaging biomarkers, Gleason score, and PSA, to predict biochemical recurrence^9^. Sometimes deep learning-based approaches even surpass the performance metrics of human-rated PI-RADS scores^18^. Compared with previous studies, the present study had several strengths. First, our study used a new deep learning framework, NAFNet, as the base architecture. NAFNet is a nonlinear activation-free network that simplifies the conventional architecture by replacing the complex nonlinear activation functions. Although simplified, its performance is equal to or better than the baseline with lower computational costs. Second, we used whole-mount histopathology of RP as the ground truth in the internal set instead of routine pathology slides used in other studies^9^. With a whole post-surgical prostate specimen provided by WSI, our radiologists could delineate the lesion more accurately on mpMRI images. Third, we independently validated our nomogram using data from other five external institutes. Results from these external testing data suggested that our model is robust and resilient to different MR scanners.

Risk stratification plays an import role in tailoring the treatment for patients with prostate cancer^19^, and artificial intelligence risk classifiers are emerging for its rapid, low-cost, and automated features^20,21^. However, while the artificial intelligence tools are evolving rapidly, the application of AI tools in medical imaging are relatively lagged due to its complexity. Various AI tools present technical problems when facing complicated clinical routine works^22^. Prostate MRI imaging is critical for prostate cancer diagnosis and follow-up, however, reading prostate MRI requires long-term experience and clinical practise for radiologist to achieve optimal results^23^. As showed in our study, the NAFNet-based integrated nomogram exhibits superior performance compared to the PI-RADS score evaluated by experienced radiologists, not only in assessing clinically significant prostate cancer but also in predicting AP and bRFS prebiopsy. This integrated nomogram holds significant promise for clinical implementation, offering the potential to alleviate the burden on radiologists. The comprehensive comparisons to CAPRA score carried out in this research emphasize the robustness and accuracy of the NAFNet-based nomogram in predicting AP and bRFS post-biopsy, highlighting the comprehensive utility and potential impact of the integrated nomogram in prostate cancer diagnosis as well as cancer management. Moreover, since the DL-nomogram demonstrated a higher net benefit in DCA curves for AP prediction, clinicians might involve patients in the decision-making process regarding aggressive treatment options by referring our DL-nomogram.

Our study had several limitations. First, we acknowledge the possibility of inter-reader variability because the ground truth in the external test set was the pathology examination from each of the institutions separately. Moreover, H2, H3, H4, H5, and H6 used ground truth data from routine pathology section images. Second, the index lesion was manually delineated in specialized software by experienced radiologists, ROI delineation at present was unavoidable. Third, BCR was used in our study as a surrogate endpoint for metastasis because of insufficient follow-up time after RP. Finally, compared to previous studies, more patients with advanced prostate cancer were included in our study to construct the deep learning model as well as the nomogram. Prior to clinical deployment, the approach needs additional external validation in the future to further confirm the generalizability in more risk groups. Currently, the interpretability of features extracted by deep learning networks is relatively weak compared with traditional machine learning based on radiomics. Therefore, NAFNet cannot extract corresponding interpretable and specific radiological features for radiologist. The interpretable deep learning networks may strike a balance between model complexity and interpretability, making them more suitable for biomedical applications. We can expect the great advancements in the field of MRI reading. The interpretable deep learning networks development is also included in our future research plan.

Despite these limitations, our study trained a robust model on a large dataset to construct a nomogram (DL-nomogram) based on a more powerful algorithm, NAFNet, integrating biopsy ISUP group, and clinical T stage to predict AP and bRFS in prostate cancer patients. With additional independent multi-site validation, the remarkable performance of DL-nomogram surpasses traditional models. This advancement holds the potential to revolutionize the pretreatment risk stratification of localized prostate cancer patients.

## Methods

### Patient cohorts

All the enrolled patients met the following criteria: patients who had (1) undergone RP after mpMRI; (2) no history of neoadjuvant, adjuvant or other additional therapy; (3) preoperative 3 Tesla (3 T) prostate MRI images of high quality; and (4) visible lesions in images.

The internal set consisted of 367 patients diagnosed between 2019 and 2021 at the Fudan University Shanghai Cancer Center (H1). All the patients in this set had whole-mount histopathology slide images (WSIs) of RP specimens, and the median follow-up time was 10 months. A second cohort of 147 patients was used as an independent external test set. These patients were diagnosed between 2017 and 2019 and were from five other tertiary hospitals: Shanghai Proton and Heavy Ion Center (H2, located in Shanghai), Ningbo No.2 Hospital (H3, outside of Shanghai), Yunnan Cancer Hospital (H4, outside of Shanghai), Jiangsu Cancer Hospital (H5, outside of Shanghai) and Henan Cancer Hospital (H6, outside of Shanghai). Patients in the external test set did not have WSIs, but they had comparatively longer follow-up period, and the median follow-up time was 32 months.

BCR was defined as at least two consecutive serum PSA levels >0.2 ng/mL after surgery^24^; biochemical recurrence-free survival (bRFS) was defined as the interval between the date of RP and the date of BCR. Patients who were still alive without BCR at the last reported follow-up were labelled as censored^9^. AP was defined as the presence of high ISUP group (ISUP ≥ 4) tumours, extensive positive surgical margin (large tumour area contacting the specimen ink)^25^, extra-prostatic extension (EPE), seminal vesicle invasion (SVI), or pelvic lymph node metastasis on the radical prostatectomy (RP) specimen as previously described^26^.

This study protocol was approved by the ethical review board of Fudan University Shanghai Cancer Center (No. 2108241-3) and was conducted in accordance with the Declaration of Helsinki. Written consent was obtained from each patient. This study was also registered in the Chinese Clinical Trial Registry (ChiCTR2000036123). The TRIPOD and STARD guidelines were also followed (Supplementary Table 6 and Supplementary Table 7).

### MRI pre-processing and lesion delineation

All patients were imaged on 3T MRI scanners with a phased-array coil, they also had axial turbo spin‒echo T2WI and axial DWI with ADC maps; detailed parameters of MRI scanners in this study are listed in Supplementary Table 8.

The MRI images were reviewed by experienced radiologists (XH.L. with 15 years of experience and ZZ.C. with 7 years of experience). PI-RADS version 2.1 (PI-RADS v2.1) scores were reassessed (if the b value was not higher than 1400, PI-RADS v2.0 was applied), and the regions of interest (ROIs) were delineated on T2WI, DWI and ADC maps using ITK-SNAP software. In order to ensure the accuracy of delineating the lesions from MRI imaging, we used whole-mount histopathology of RP specimens’ images to delineate the extent of lesions on MRI images, and used them as ROIs. Besides, the pathology results from whole-mount histopathology of RP specimens were treated as the gold standard of AP. To construct the deep learning model more precisely, a senior urological pathologist (YY.K.) was invited to label the slides with AP and mark the index lesion (defined as the largest lesion, the highest suspicion based on images, or the tumour with the highest grade) on the whole-mount histopathology RP specimens for each patient^27,28^; then, a senior radiologist (XH.L.) identified tumour index lesion regions on T2WI, ADC and DWI MRI imaging using the marked slides as a reference in the training set. In the external test set, the suspicious lesion was determined by study radiologists who were blinded to the pathology report.

After MRI image input, we then normalized MRI images, which involved scaling the image values to the range of 0 to 1 by dividing them by the maximum value of the image. Of all three channels, 2D MRI images were used. Next, we resized the images to the same dimensions for ease of model processing, and applied data augmentation techniques including flipping and rotating to increase the diversity of samples. All of these steps were taken to enhance the performance and robustness of the model.

### Construction of NAFNet classifier and comparison to ResNet50 in predicting AP

Nonlinear Activation Free Network, NAFNet, is a newly developed deep learning network originally proposed by the co-author of our study, LY.C. in 2022^15^. NAFNet achieved state-of-the-art results in the field of image restoration, e.g., denoising, deblurring, and stereo super-resolution^29^. The original intention behind the design of NAFNet is to address pixel-level issues. Firstly, NAFNet avoids the pixel accuracy problems caused by batch normalization and instead uses pixel-level layer normalization. Additionally, the U-Net structure and skip connections in NAFNet allow for better information transfer from input to output, thus avoiding accuracy degradation due to information loss. Thus, the dense prediction (pixelwise prediction) of NAFNet allows it to cut the features in the ROI more precisely, offering promising potential in MRI classification. The original Python codes of NAFNet are available on https://github.com/megvii-research/NAFNet. Compared to NAFNet, ResNet50, a widely-used residual networks developed by He at in 2015^14^, has a deep architecture with 50 convolutional layers, divided into several modules, each containing residual blocks of different depths. This design allows ResNet50 to better propagate gradients, improve network convergence, and accuracy, and suffered from more complicated network design.

NAFNet comprised three distinct input channels: T2WI, ADC and DWI maps. The image augmentation strategies and optimized configuration were as follows: we adopted random flipping/rotation and resized the maps to a spatial size of 384×384 by bilinear interpolation. The network was optimized using an AdamW optimizer, with a weight decay of 0.05 and a learning rate of 0.00001.

We labelled AP events as 1 and no AP events as 0. Training and validation on the internal set were performed patient-wise using threefold repeated validation with an 80% (training) to 20% (validation) stratified, random split for each fold. The results derived from the highest value of the cross-validation area under the curve (AUC) of the receiver operating characteristic (ROC) curve from the validation set were chosen to evaluate performance on the external test set. A binary entropy loss function was used in the deep learning network.

Firstly, we classified each pixel within the ROI region of the input image. Then, we aggregated the scores of all pixels in the ROI region to obtain the overall classification result of the entire image. Specifically, the input image and ROI region are fed into the network, which outputs features with the same size as the image. Then, these features are passed through a fully connected layer to obtain a single-channel value, which is further transformed into a binary probability using the Sigmoid function. In this way, we obtain pixel-level probability. The final probability is determined by the pixel-level probabilities in the ROI region. After averaging the probability values of each patient’s three channels’ images, the final probability for that patient is obtained.

All deep learning experiments were performed in the Python framework (Python 3.9.5, PyTorch 1.11.0 and CUDA 11.3). All models were trained with the same hardware configuration (CPU with 16 cores and 64 GB RAM; one GPU of Nvidia A100 with 40 GB of memory), supported by the Medical Science Data Center of Fudan University.

### Construction of deep-learning nomogram

Multivariable logistic regression models were fitted with a deep learning-based image classifier (NAFNet-classifier) and various clinicopathologic parameters, including preoperative PSA, biopsy ISUP, Prostate Imaging Reporting and Data System (PI-RADS) score, and clinical stage (cT), to evaluate the prediction of AP after RP. We treated NAFNet-classifier as a continuous variable and included it in the multivariable logistic regression model along with other clinicopathological factors, such as PI-RADS score, biopsy ISUP, cT stage and PSA value. We use the enter method for regression model. After calculation, factors with statistically significant *P*-values were selected for further nomogram construction.

### Clinical validation of DL-nomogram to predict AP, bRFS and clinically significant prostate cancer prebiopsy

The CAPRA score (including age, PSA, Gleason score of the biopsy, clinical T stage and percent of biopsy cores involved with cancer) is one of the most widely used model to predict AP, risk of BCR and metastasis, and prostate cancer-specific survival^30^. The Prostate Biopsy Collaborative Group (PBCG) risk score (including race, age, PSA, digital rectal examination, prior biopsy and family history of prostate cancer) is used to predict the risk of high-grade prostate cancer before prostate biopsy^31^. We used CAPRA score and PBCG score as the clinical score and the PI-RADS score as the radiology score when performing comparisons. In summary, we performed three further analyses to clinically validate the performance of NAFNet-classifier and DL-nomogram: (1) comparison of DL-nomogram with the radiology score (PI-RADS) and the clinical score (CAPRA) in the prediction of AP; (2) a head-to-head comparison between NAFNet-classifier, DL-nomogram, the radiology score (PI-RADS) and the clinical score (CAPRA) for bRFS prediction in terms of the concordance index (C-index) and Kaplan‒Meier curves; and (3) comparison of NAFNet-classifier and DL-nomogram with the clinical score (PBCG) in the prediction of clinically significant prostate cancer prebiopsy.

### Statistical analysis

Significant differences in areas under the curve (AUCs) between the models were tested using DeLong’s test^32^. We report the AUCs with 95% CIs and other performance metrics (including accuracy, sensitivity and specificity) in the external test set. The 95% CI was calculated based on the R package “pROC” with “delong” methods^32^ to obtain the 95%CI of AUC following the documentation of “pROC” package^33^. Multivariable analyses were used to develop the nomograms. Decision curve analyses (DCAs) were used to illustrate the overall net benefit of using one model versus another. Kaplan‒Meier survival curves were used for bRFS analysis. In order to achieve the best performance of the binary classification, we obtained the optimal cut-off points by maximizing the accuracy in the ROC curves of each model as previously described^17^. The log-rank test was used in bRFS to determine statistically significant differences. All tests were two-sided, and statistical significance was defined as *P* < 0.05. Statistical analyses were performed using R software version 4.2.1.

### Reporting summary

Further information on research design is available in the Nature Research Reporting Summary linked to this article.

### Supplementary information

Supplementary Information

Reporting Summary

## Data Availability

Requests to original datasets should be made directly via corresponding author (M.D. Bo Dai, E-mail: bodai1978@126.com) with a data access request form, institute rules and regulation of data access should be followed.

## Code availability

All code was implemented in Python 3.9 and R 4.3.1. Original NAFNet model code is available on https://github.com/megvii-research/NAFNet. Custom Python and R code related to this study is publicly available on https://github.com/SHMCLz/NAFNet-for-Prostate-MRI.
