# Supplementary material for: A deep learning model, NAFNet, predicts adverse pathology and recurrence in prostate cancer using MRIs
Source: NPJ Precis Oncol. 2023 Dec 11;7:134. doi: 10.1038/s41698-023-00481-x (PMC10713594; doi:10.1038/s41698-023-00481-x)
Supplement: Supplementary file 1 — Supplementary Information [file 41698_2023_481_MOESM1_ESM.pdf]

Supplementary Information

Supplementary Tables:

Supplementary Table 1. Performance comparisons between different models in adverse pathology prediction on test set

Supplementary Table 2. Confusion matrices comparisons among different models in adverse pathology prediction on test set

Supplementary Table 3. Model comparisons between different models in biochemical recurrence-free survival prediction

Supplementary Table 4. Multivariate Logistics regression analyses of clinicopathological features and NAFNet-classifier for ISUP/Gleason grade prediction

Supplementary Table 5. Performance comparisons between different models in postsurgical ISUP high risk group (≥4) prediction

Supplementary Table 6. TRIPOD Checklist of this study

Supplementary Table 7. Checklist for STARD of this study

Supplementary Table 8. Main parameters of MRI acquisition in this study

Supplementary Figures:

Supplementary Figure 1. Confusion matrices comparison of AP predicting models at dichotomous setting

Supplementary Figure 2. Calibration plot of the DL-nomogram model

Supplementary Figure 3. Performance comparison of continuous NAFNet-classifier and dichotomous NAFNet-classifier

Supplementary Figure 4. Construction and performance analysis of various models based on the NAFNet-classifier in predicting adverse pathology events

Supplementary Figure 5. Performance analysis of various models in predicting post-surgical ISUP ≥4 group.

Supplementary Tables

Supplementary Table 1. Performance comparisons between different models in adverse pathology prediction on test set

Model	AUC on test set (95%CI)	Delong's Test *P* for comparing AUCs				Accuracy	Sensitivity	Specificity	Threshold
DL-nomogram	0.915 (0.871-0.959)	<0.001	<0.001	0.009	Reference	0.850	0.852	0.848	0.370
ResNet50-calssifer	0.703 (0.618-0.787)	0.385	0.947	0.013	<0.001	0.687	0.704	0.667	0.326
NAFNet-classifier	0.798 (0.724-0.873)	0.334	0.025	Reference		0.769	0.827	0.697	0.389
Radiology score (PI-RADS)	0.699 (0.621-0.777)	0.256	Reference			0.680	0.728	0.621	2.500
Clinical score (CAPRA)	0.751 (0.679-0.822)	Reference				0.734	0.765	0.697	2.500
DL-nomogram plus PSA	0.913 (0.869-0.958)				0.791	0.837	0.852	0.818	0.355
DL-nomogram plus PI-RADS	0.912 (0.868-0.957)				0.194	0.843	0.827	0.864	0.380
DL-nomogram plus PSA and PI-RADS	0.913 (0.868-0.957)				0.685	0.837	0.827	0.848	0.373

AUC: area under the receiver operating characteristic curve; CI: confidence interval; DL: Deep learning; PI-RADS: Prostate Imaging–Reporting & Data System; CAPRA: Prostate Cancer Risk Assessment; PSA: prostate specific antigen;

Supplementary Table 2. Confusion matrices comparisons among different models in adverse pathology prediction on test set

Models on test set	Accuracy (95%CI)	Sensitivity	Specificity	Positive prediction value	Negative prediction value
dichotomous NAFNet-classifier	0.769 (0.692-0.834)	0.827	0.697	0.770	0.767
dichotomous ResNet50-classifier	0.687 (0.605-0.761)	0.704	0.667	0.721	0.647
dichotomous DL-nomogram	0.850 (0.782-0.904)	0.852	0.848	0.873	0.823
dichotomous radiology score (PI-RADS)	0.683 (0.598-0.755)	0.728	0.621	0.702	0.651
dichotomous clinical score (CAPRA)	0.738 (0.656-0.804)	0.765	0.697	0.756	0.707

CI: confidence interval; DL: Deep learning; PI-RADS: Prostate Imaging–Reporting & Data System; CAPRA: Prostate Cancer Risk Assessment;

Supplementary Table 3. Model comparisons between different models in biochemical recurrence-free survival prediction

Model	c-index (95%CI)	*P* value for comparing c-index*		
DL-nomogram	0.732 (0.671-0.793)	<0.001	<0.001	<0.001
NAFNet-classifier	0.635 (0.566-0.703)	<0.001	<0.001	Reference
Radiology score (PI-RADS)	0.630 (0.571-0.689)	<0.001	Reference	
Clinical score (CAPRA)	0.643 (0.586-0.700)	Reference		

*ANOVA test was applied.

CI: confidence interval; DL: Deep-learning; PI-RADS: Prostate Imaging–Reporting & Data System; CAPRA: Prostate Cancer Risk Assessment;

Supplementary Table 4. Multivariate Logistics regression analyses of clinicopathological features and NAFNet-classifier for ISUP/Gleason grade prediction

Factor	OR (95% CI)	*P*
For postsurgical ISUP high risk group (≥4)		
NAFNet-classifier*	1.034 (1.024-1.044)	<0.001
PI-RADS score	1.305 (0.944-1.821)	0.111
Clinical T stage (≥cT3 vs. cT2)	3.602 (1.849-7.311)	<0.001
PSA at diagnosis, ng/ml (≥20 vs. <20)	2.182 (1.387-3.428)	<0.001

* At every 1% DL-classifier

ISUP: International Society of Urological Pathology; OR: Odds Ratio; CI: confidence interval; PI-RADS: Prostate Imaging–Reporting & Data System; PSA: prostate specific antigen.

Supplementary Table 5. Performance comparisons between different models in postsurgical ISUP high risk group (≥4) prediction

Model	AUC on all set (95%CI)	Delong's Test *P* for comparing AUCs			Accuracy	Sensitivity	Specificity	Threshold
DLPT-nomogram	0.805 (0.766-0.844)	0.003	<0.001	0.399	0.753	0.663	0.802	0.405
NAFNet-classifier	0.781 (0.740-0.821)	0.006	<0.001	Reference	0.669	0.811	0.596	0.453
Radiology score (PI-RADS)	0.642 (0.601-0.683)	0.008	Reference		0.589	0.766	0.499	2.500
Clinical score (PBCG)	0.714 (0.667-0.760)	Reference			0.650	0.731	0.608	0.544

ISUP: International Society of Urological Pathology; AUC: area under the receiver operating characteristic curve; CI: confidence interval; PI-RADS: Prostate Imaging–Reporting & Data System; PBCG: Prostate Biopsy Collaborative Group score;

Supplementary Table 6. TRIPOD Checklist of this study

Section/Topic	Item		Checklist Item	Page
**Title and abstract**				
Title	1	D;V	Identify the study as developing and/or validating a multivariable prediction model, the target population, and the outcome to be predicted.	NA
Abstract	2	D;V	Provide a summary of objectives, study design, setting, participants, sample size, predictors, outcome, statistical analysis, results, and conclusions.	1
**Introduction**				
Background and objectives	3a	D;V	Explain the medical context (including whether diagnostic or prognostic) and rationale for developing or validating the multivariable prediction model, including references to existing models.	1
	3b	D;V	Specify the objectives, including whether the study describes the development or validation of the model or both.	1
**Methods**				
Source of data	4a	D;V	Describe the study design or source of data (e.g., randomized trial, cohort, or registry data), separately for the development and validation data sets, if applicable.	7
	4b	D;V	Specify the key study dates, including start of accrual; end of accrual; and, if applicable, end of follow-up.	7
Participants	5a	D;V	Specify key elements of the study setting (e.g., primary care, secondary care, general population) including number and location of centres.	7
	5b	D;V	Describe eligibility criteria for participants.	7
	5c	D;V	Give details of treatments received, if relevant.	7
Outcome	6a	D;V	Clearly define the outcome that is predicted by the prediction model, including how and when assessed.	8
	6b	D;V	Report any actions to blind assessment of the outcome to be predicted.	8
Predictors	7a	D;V	Clearly define all predictors used in developing or validating the multivariable prediction model, including how and when they were measured.	8
	7b	D;V	Report any actions to blind assessment of predictors for the outcome and other predictors.	8
Sample size	8	D;V	Explain how the study size was arrived at.	7
Missing data	9	D;V	Describe how missing data were handled (e.g., complete-case analysis, single imputation, multiple imputation) with details of any imputation method.	Fig 1a
Statistical analysis methods	10a	D	Describe how predictors were handled in the analyses.	8
	10b	D	Specify type of model, all model-building procedures (including any predictor selection), and method for internal validation.	8
	10c	V	For validation, describe how the predictions were calculated.	8
	10d	D;V	Specify all measures used to assess model performance and, if relevant, to compare multiple models.	8
	10e	V	Describe any model updating (e.g., recalibration) arising from the validation, if done.	8
Risk groups	11	D;V	Provide details on how risk groups were created, if done.	NA
Development vs. validation	12	V	For validation, identify any differences from the development data in setting, eligibility criteria, outcome, and predictors.	7
**Results**				
Participants	13a	D;V	Describe the flow of participants through the study, including the number of participants with and without the outcome and, if applicable, a summary of the follow-up time. A diagram may be helpful.	Fig 1a
	13b	D;V	Describe the characteristics of the participants (basic demographics, clinical features, available predictors), including the number of participants with missing data for predictors and outcome.	Table 1
	13c	V	For validation, show a comparison with the development data of the distribution of important variables (demographics, predictors and outcome).	Table 1
Model development	14a	D	Specify the number of participants and outcome events in each analysis.	Table 1
	14b	D	If done, report the unadjusted association between each candidate predictor and outcome.	2
Model specification	15a	D	Present the full prediction model to allow predictions for individuals (i.e., all regression coefficients, and model intercept or baseline survival at a given time point).	Fig 3
	15b	D	Explain how to the use the prediction model.	5-6
Model performance	16	D;V	Report performance measures (with CIs) for the prediction model.	Fig2-4
Model-updating	17	V	If done, report the results from any model updating (i.e., model specification, model performance).	Table S1, S2
**Discussion**				
Limitations	18	D;V	Discuss any limitations of the study (such as nonrepresentative sample, few events per predictor, missing data).	6
Interpretation	19a	V	For validation, discuss the results with reference to performance in the development data, and any other validation data.	5-6
	19b	D;V	Give an overall interpretation of the results, considering objectives, limitations, results from similar studies, and other relevant evidence.	5
Implications	20	D;V	Discuss the potential clinical use of the model and implications for future research.	5-6
**Other information**				
Supplementary information	21	D;V	Provide information about the availability of supplementary resources, such as study protocol, Web calculator, and data sets.	9
Funding	22	D;V	Give the source of funding and the role of the funders for the present study.	9

*Items relevant only to the development of a prediction model are denoted by D, items relating solely to a validation of a prediction model are denoted by V, and items relating to both are denoted D;V.

Supplementary Table 7. Checklist for STARD of this study			
**Section & Topic**	**No**	**Item**	**Reported on page #**
**TITLE OR ABSTRACT**			
	**1**	Identification as a study of diagnostic accuracy using at least one measure of accuracy (such as sensitivity, specificity, predictive values, or AUC)	1
**ABSTRACT**			
	**2**	Structured summary of study design, methods, results, and conclusions (for specific guidance, see STARD for Abstracts)	1
**INTRODUCTION**			
	**3**	Scientific and clinical background, including the intended use and clinical role of the index test	1
	**4**	Study objectives and hypotheses	1
**METHODS**			
Study design	**5**	Whether data collection was planned before the index test and reference standard were performed (prospective study) or after (retrospective study)	2
Participants	**6**	Eligibility criteria	Fig 1a
	**7**	On what basis potentially eligible participants were identified (such as symptoms, results from previous tests, inclusion in registry)	Fig 1a
	**8**	Where and when potentially eligible participants were identified (setting, location and dates)	7
	**9**	Whether participants formed a consecutive, random or convenience series	7
Test methods	**10a**	Index test, in sufficient detail to allow replication	7-8
	**10b**	Reference standard, in sufficient detail to allow replication	7-8
	**11**	Rationale for choosing the reference standard (if alternatives exist)	7-8
	**12a**	Definition of and rationale for test positivity cut-offs or result categories of the index test, distinguishing pre-specified from exploratory	8
	**12b**	Definition of and rationale for test positivity cut-offs or result categories of the reference standard, distinguishing pre-specified from exploratory	8
	**13a**	Whether clinical information and reference standard results were available to the performers/readers of the index test	8
	**13b**	Whether clinical information and index test results were available to the assessors of the reference standard	8
Analysis	**14**	Methods for estimating or comparing measures of diagnostic accuracy	8
	**15**	How indeterminate index test or reference standard results were handled	8
	**16**	How missing data on the index test and reference standard were handled	Fig 1a
	**17**	Any analyses of variability in diagnostic accuracy, distinguishing pre-specified from exploratory	Table S1
	**18**	Intended sample size and how it was determined	Fig 1a
**RESULTS**			
Participants	**19**	Flow of participants, using a diagram	Fig 1a
	**20**	Baseline demographic and clinical characteristics of participants	Table 1
	**21a**	Distribution of severity of disease in those with the target condition	NA
	**21b**	Distribution of alternative diagnoses in those without the target condition	NA
	**22**	Time interval and any clinical interventions between index test and reference standard	7
Test results	**23**	Cross tabulation of the index test results (or their distribution) by the results of the reference standard	Table 1
	**24**	Estimates of diagnostic accuracy and their precision (such as 95% confidence intervals)	Fig 2-4
	**25**	Any adverse events from performing the index test or the reference standard	NA
**DISCUSSION**			
	**26**	Study limitations, including sources of potential bias, statistical uncertainty, and generalisability	6
	**27**	Implications for practice, including the intended use and clinical role of the index test	5-6
**OTHER INFORMATION**			
	**28**	Registration number and name of registry	7
	**29**	Where the full study protocol can be accessed	9
	**30**	Sources of funding and other support; role of funders	9

Supplementary Table 8. Main parameters of MRI acquisition in this study

**Cohort**	**H1 (n=367)**			**H2 (n=100)**		**H3 (n=10)**	**H4 (n=12)**	**H5 (n=15)**	**H6 (n=10)**
**Scanner**									
Manufacturer	GE	Siemens	GE	GE	Siemens	Simens	Philips	Philips	Siemens
Type	HDxt 3T	skyra	signa architect	signa pioneer	skyra	prisma	Ingenia	Ingenia	skyra
Coil type	phased-array coil	phased-array coil	phased-array coil	phased-array coil	phased-array coil	phased-array coil	phased-array coil	phased-array coil	phased-array coil
Number of Subjects	169	182	16	73	27	10	12	15	10
**T2-weighted MRI**									
Field-of-view (mm^2)	256 * 256	160 * 160	180 * 180	220 * 220	240 * 240	200 * 200	200 * 200	280 * 280	220 * 220
Matrix size	512 * 512	320 * 320	512 * 512	512 * 512	320 * 320	320 * 320	480 * 480	512 * 512	384 * 384
TR (repetition time, ms)	2980	9040	6035	3621	9040	6000	2235	2789	6600
TE (echo time, ms)	107	89	89.4	93	89	101	90	100	82
Acquisition time (min)	2.0	2.0	2.0	2.0	2.0	1.9	2.0	2.86	2.60
**Diffusion-weighted MRI (ADC derived from DWI)**									
Field-of-view (mm^2)	260 * 260	134 * 29	180 * 180	200 * 200	200 * 200	100 * 180	200 * 200	200 * 200	219 * 219
Matrix size	256 * 256	116 * 88	256 * 256	256 * 256	116 * 116	100 * 180	112 * 112	128 * 128	142 * 142
TR (repetition time, ms)	4000	6680	5700	5097	6680	3800	4339	6000	3900
TE (echo time, ms)	72	116	80.9	74.5	58	70	73	78	56
Acquisition time (min)	2.5	5.0	5.0	5.0	2.0	1.86	2.0	2.87	2.68
b-value	0, 1000	0, 1500	0, 1500	0, 1000, 1500	0, 1000,	0,50,1500	0, 1500	0, 1200	0,800

Supplementary Figures

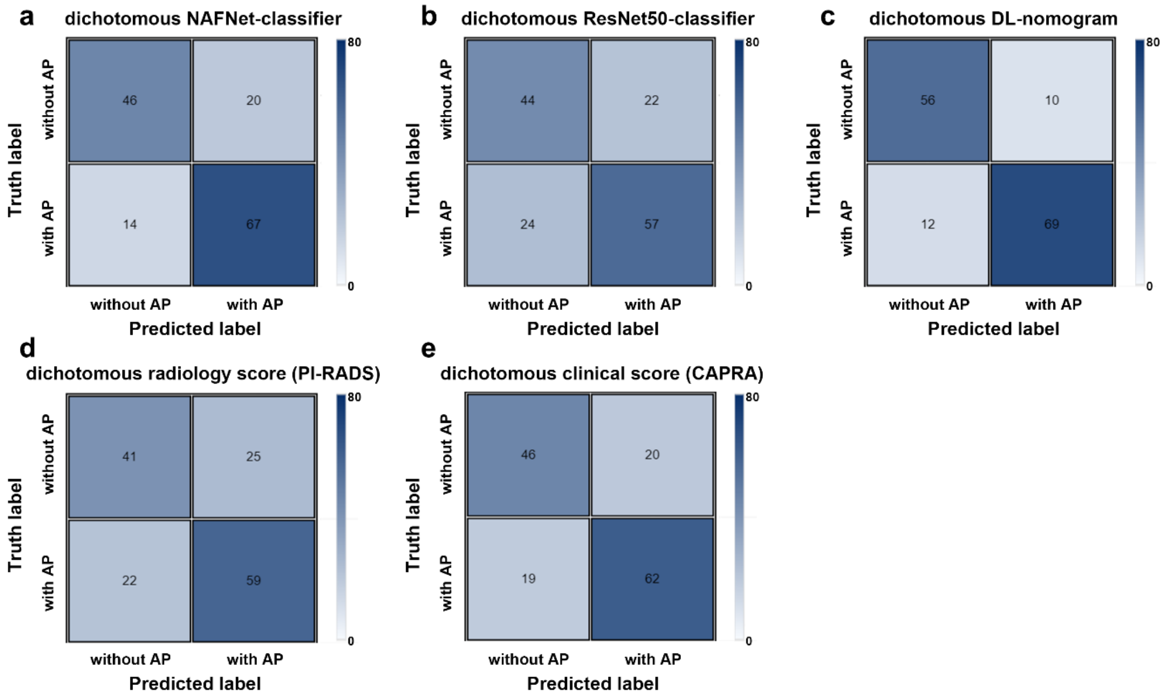

Supplementary Figure 1. Confusion matrices comparison of AP predicting models at dichotomous setting

Confusion matrices of (a) dichotomous NAFNet-classifier, (b) dichotomous ResNet50-classifer, (c) dichotomous DL-nomogram, (d) dichotomous radiology score (PI-RADS), and (e) dichotomous clinical score (CAPRA) in predicting AP on test sets. Threshold of each model was obtained by maximizing the accuracy in the ROC curves of corresponding model, and detailed threshold point was listed in Supplementary Table 2. Abbreviations: AP: adverse pathology; DL: deep learning; PI-RADS: Prostate Imaging–Reporting and Data System; CAPRA: Prostate Cancer Risk Assessment; AUC: area under the receiver operating characteristic curve; CI: confidence interval.

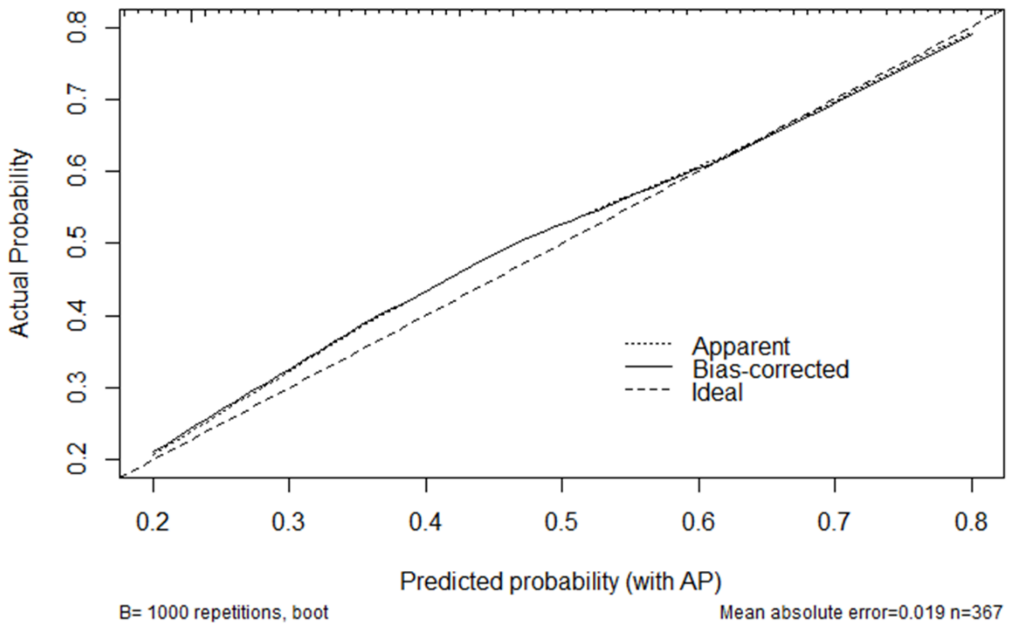

Supplementary Figure 2. Calibration plot of the DL-nomogram model

The calibration plot of the DL-nomogram. Bootstrap n=1000 analysis was applied to avoid overfitting. Abbreviations: AP: adverse pathology; DL: deep learning.

a

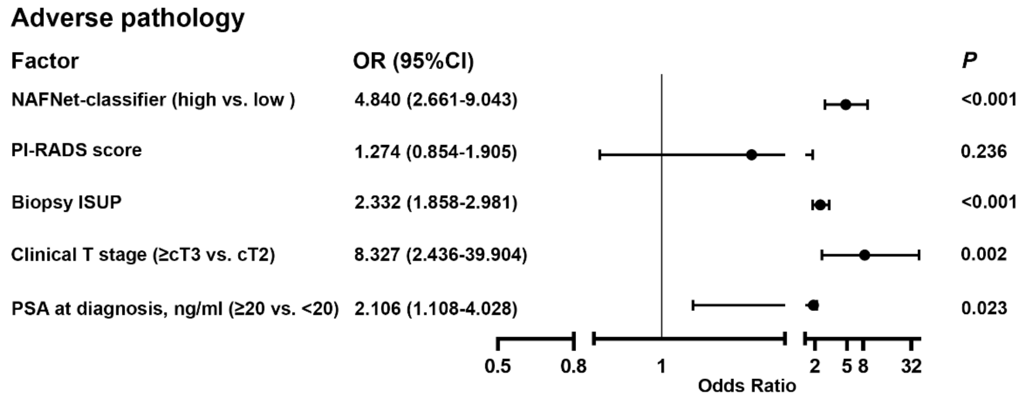

b

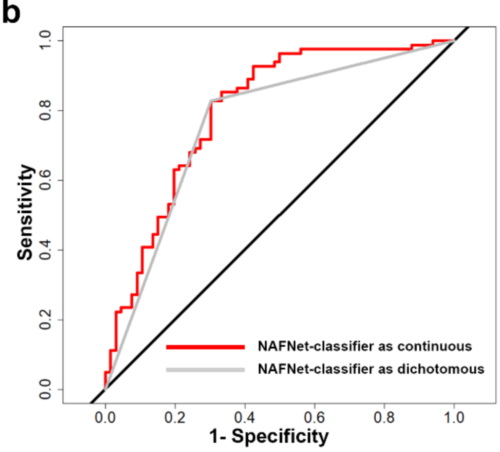

c

**Models**	**AUC on test set (95%CI)**	**Delong's Test *P***
NAFNet-classifier as continuous	0.798 (0.724-0.873)	Reference
NAFNet-classifier as dichotomous	0.762 (0.692-0.832)	0.077

Supplementary Figure 3. Performance comparison of continuous NAFNet-classifier and dichotomous NAFNet-classifier

(a) Forest plot shows the multivariate logistic regression analyses constructed based on internal set for predicting adverse pathology events. NAFNet-classifier was treated as dichotomous variable. Receiver operating characteristics curve (b) and AUC comparison (c) of continuous NAFNet-classifier (red) and dichotomous NAFNet-classifier (grey) in predicting adverse pathology events based on external test set. Abbreviations: PI-RADS: Prostate Imaging–Reporting and Data System; ISUP: International Society of Urological Pathology; PSA: prostate specific antigen; OR: odds ratio; CI: confidence interval; AUC: area under the receiver operating characteristic curve.

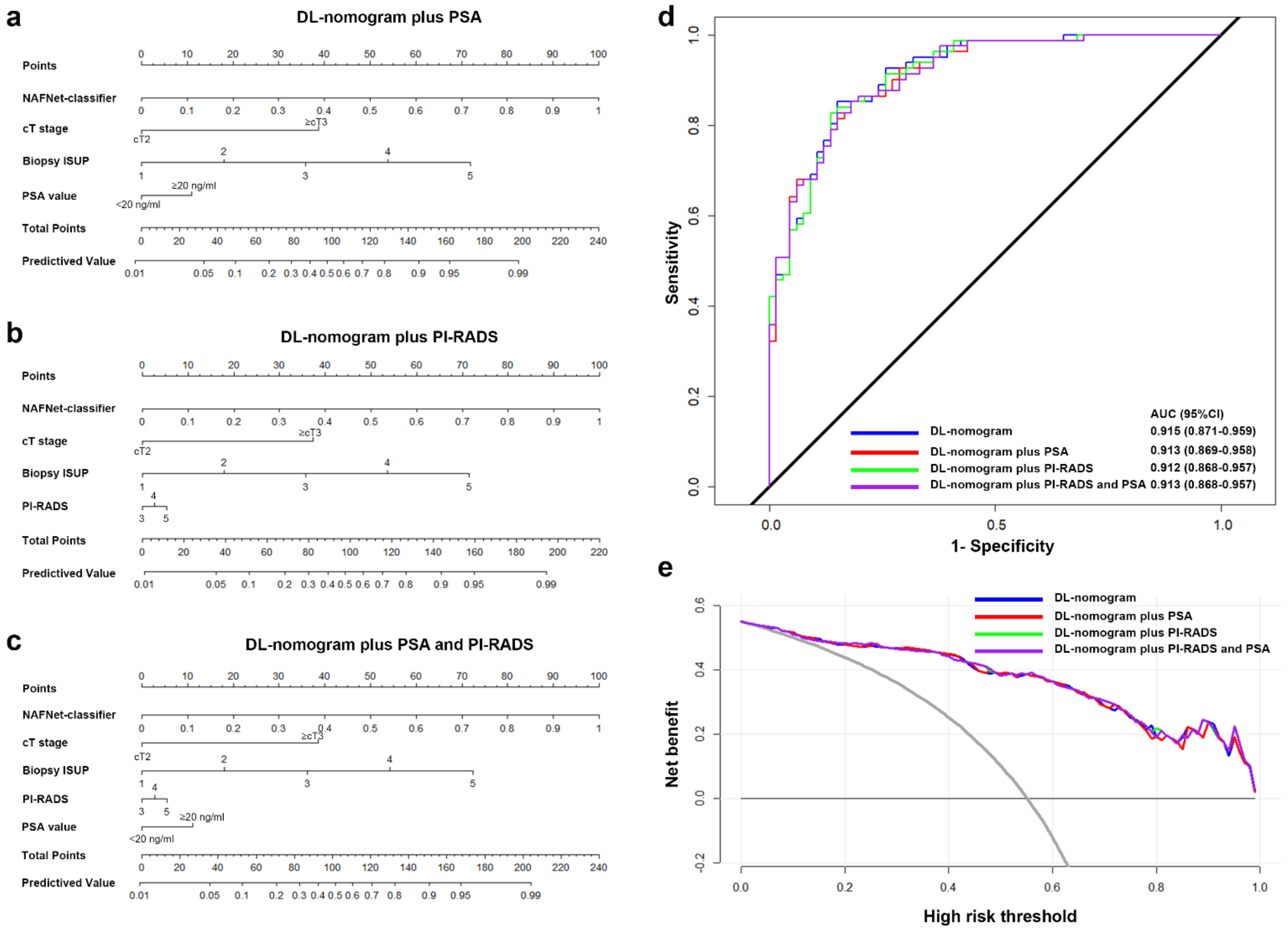

Supplementary Figure 4. Construction and performance analysis of various models based on the NAFNet-classifier in predicting adverse pathology events

(a) A nomogram combining the NAFNet-classifier, cT stage, biopsy ISUP and PSA value. (b) A nomogram combining the NAFNet-classifier, cT stage, biopsy ISUP and PI-RADS score. (c) A nomogram combining the NAFNet-classifier, cT stage, biopsy ISUP, PSA value and PI-RADS score. (d) Receiver operating characteristics curves and (e) decision curve analyses of the DL-nomogram, DL-nomogram plus PSA, DL-nomogram plus PI-RADS and DL-nomogram plus PI-RADS and PSA model in predicting adverse pathology events. Abbreviations:

DL: deep learning; PSA: prostate specific antigen; PI-RADS: Prostate Imaging–Reporting and Data System; AUC: area under the receiver operating characteristic curve; CI: confidence interval.

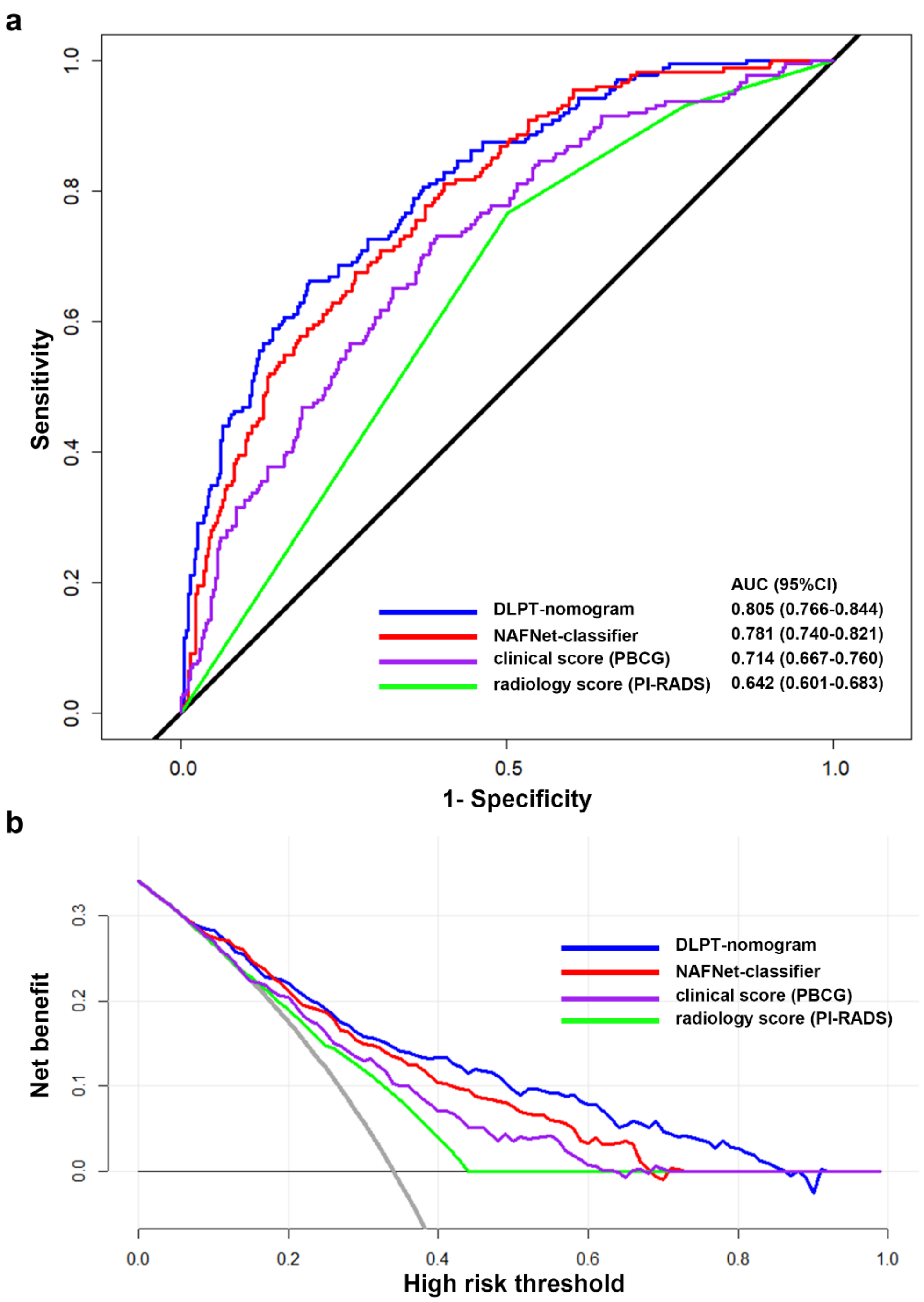

Supplementary Figure 5. Performance analysis of various models in predicting post-surgical ISUP ≥4 group.

(a) Receiver operating characteristics curves of the DLPT-nomogram, NAFNet-classifier, radiology score (PI-RADS), and clinical score (PBCG) in predicting the post-surgical ISUP ≥4 group. (b) Decision curve analyses of the DLPT-

nomogram, NAFNet-classifier, radiology score (PI-RADS), and clinical score (PBCG) in predicting the post-surgical ISUP ≥4 group. Abbreviations: DLPT: NAFNet-classifier integrated with PSA and cT stage; DL: deep learning; PI-RADS: Prostate Imaging–Reporting and Data System; PBCG: Prostate Biopsy Collaborative Group model; AUC: area under the receiver operating characteristic curve; CI: confidence interval.
