# Supplementary material for: A deep learning model, NAFNet, predicts adverse pathology and recurrence in prostate cancer using MRIs
Source: NPJ Precis Oncol. 2023 Dec 11;7:134. doi: 10.1038/s41698-023-00481-x (PMC10713594; doi:10.1038/s41698-023-00481-x)
Supplement: Supplementary file 2 — Reporting Summary [file 41698_2023_481_MOESM2_ESM.pdf]

Corresponding author(s): Bo Dai
Last updated by author(s): 2023.11.11

# Reporting Summary

Nature Portfolio wishes to improve the reproducibility of the work that we publish. This form provides structure for consistency and transparency in reporting. For further information on Nature Portfolio policies, see our Editorial Policies and the Editorial Policy Checklist.

## Statistics

For all statistical analyses, confirm that the following items are present in the figure legend, table legend, main text, or Methods section.

| n/a | Confirmed | |
|---|---|---|
| ☐ | ☒ | The exact sample size (*n*) for each experimental group/condition, given as a discrete number and unit of measurement |
| ☐ | ☒ | A statement on whether measurements were taken from distinct samples or whether the same sample was measured repeatedly |
| ☐ | ☒ | The statistical test(s) used AND whether they are one- or two-sided<br>*Only common tests should be described solely by name; describe more complex techniques in the Methods section.* |
| ☐ | ☒ | A description of all covariates tested |
| ☐ | ☒ | A description of any assumptions or corrections, such as tests of normality and adjustment for multiple comparisons |
| ☐ | ☒ | A full description of the statistical parameters including central tendency (e.g. means) or other basic estimates (e.g. regression coefficient) AND variation (e.g. standard deviation) or associated estimates of uncertainty (e.g. confidence intervals) |
| ☐ | ☒ | For null hypothesis testing, the test statistic (e.g. *F*, *t*, *r*) with confidence intervals, effect sizes, degrees of freedom and *P* value noted<br>*Give P values as exact values whenever suitable.* |
| ☒ | ☐ | For Bayesian analysis, information on the choice of priors and Markov chain Monte Carlo settings |
| ☒ | ☐ | For hierarchical and complex designs, identification of the appropriate level for tests and full reporting of outcomes |
| ☒ | ☐ | Estimates of effect sizes (e.g. Cohen's *d*, Pearson's *r*), indicating how they were calculated |

*Our web collection on statistics for biologists contains articles on many of the points above.*

## Software and code

Policy information about availability of computer code

| | |
|---|---|
| Data collection | Original Python codes of NAFNet are available on https://github.com/megvii-research/NAFNet. Custom python code and R code for this study is available on https://github.com/SHMCLz/NAFNet-for-Prostate-MRI. |
| Data analysis | All tests were two-sided, and statistical significance was defined as P<0.05. Statistical analyses were performed using R software version 4.2.1. |

For manuscripts utilizing custom algorithms or software that are central to the research but not yet described in published literature, software must be made available to editors and reviewers. We strongly encourage code deposition in a community repository (e.g. GitHub). See the Nature Portfolio guidelines for submitting code & software for further information.

## Data

Policy information about availability of data

All manuscripts must include a data availability statement. This statement should provide the following information, where applicable:

- Accession codes, unique identifiers, or web links for publicly available datasets
- A description of any restrictions on data availability
- For clinical datasets or third party data, please ensure that the statement adheres to our policy

Requests to original datasets should be made directly via corresponding author (M.D. Bo Dai, E-mail: bodai1978@126.com) with a data access request form, institute rules and regulation of data access should be followed.

## Research involving human participants, their data, or biological material

Policy information about studies with human participants or human data. See also policy information about sex, gender (identity/presentation), and sexual orientation and race, ethnicity and racism.

| | |
|---|---|
| Reporting on sex and gender | All prostate cancer patients involved in this study are male. |
| Reporting on race, ethnicity, or other socially relevant groupings | Not applicable in this study. |
| Population characteristics | Detailed patient characteristics were listed in Table 1 |
| Recruitment | Inclusion and exclusion criteria were illustrated in Figure 1a |
| Ethics oversight | This study protocol was approved by the ethical review board of Fudan University Shanghai Cancer Center (No. 2108241-3) and was conducted in accordance with the Declaration of Helsinki. Written consent was obtained from each patient. |

Note that full information on the approval of the study protocol must also be provided in the manuscript.

# Field-specific reporting

Please select the one below that is the best fit for your research. If you are not sure, read the appropriate sections before making your selection.

☒ Life sciences ☐ Behavioural & social sciences ☐ Ecological, evolutionary & environmental sciences

For a reference copy of the document with all sections, see nature.com/documents/nr-reporting-summary-flat.pdf

# Life sciences study design

All studies must disclose on these points even when the disclosure is negative.

| | |
|---|---|
| Sample size | Sample size was limited due to the institutional data. We retrospectively enrolled 618 patients with prostate cancer from six institutes throughout China. As depicted in Figure 1A, after screening, 367 eligible patients from Fudan University Shanghai Cancer Center were allocated to the internal set. The external test set consisted of 147 patients from other five external institutes. |
| Data exclusions | As shown in Figure 1a, Exclusion criterion includes (1) MRI with incomplete sequence, (2) MRI scan with severe artifacts, (3) Incomplete information of clinicopathologic factors. |
| Replication | Not applicable |
| Randomization | In Internal set, patients were randomized into the training set (n=294, 80%) and validation set(n=73, 20%). |
| Blinding | The suspicious lesion was determined by study radiologists who were blinded to the pathology report. |

# Reporting for specific materials, systems and methods

We require information from authors about some types of materials, experimental systems and methods used in many studies. Here, indicate whether each material, system or method listed is relevant to your study. If you are not sure if a list item applies to your research, read the appropriate section before selecting a response.

### Materials & experimental systems

| n/a | Involved in the study | |
|---|---|---|
| ☒ | ☐ | Antibodies |
| ☒ | ☐ | Eukaryotic cell lines |
| ☒ | ☐ | Palaeontology and archaeology |
| ☒ | ☐ | Animals and other organisms |
| ☒ | ☐ | Clinical data |
| ☒ | ☐ | Dual use research of concern |
| ☒ | ☐ | Plants |

### Methods

| n/a | Involved in the study | |
|---|---|---|
| ☒ | ☐ | ChIP-seq |
| ☒ | ☐ | Flow cytometry |
| ☒ | ☐ | MRI-based neuroimaging |
